# Subclinical depressive symptoms and job stress differentially impact memory in working and retired older adults

**DOI:** 10.1038/s41598-025-87333-9

**Published:** 2025-01-25

**Authors:** Lorena A. Ferguson, Amritha Harikumar, Stephanie L. Leal

**Affiliations:** 1 Department of Psychological Sciences, Rice University, 6100 Main St, Houston, TX 77005 USA; 2 Department of Integrative Biology & Physiology, UCLA, 621 Charles E Young Dr S, Los Angeles, CA 90095 USA

**Keywords:** Human behaviour, Cognitive ageing

## Abstract

Retirement has been associated with cognitive decline beyond normal age-related decline. However, there are many individual differences in retirement that can influence cognition. Subclinical depressive symptoms are common in late life and are associated with general memory decline and a bias towards remembering negative events (i.e., better memory for negative vs. positive or neutral stimuli), in opposition to a reported positivity bias (i.e., better memory for positive vs. negative or neutral stimuli) in aging. Furthermore, job stress is often a major contributor to retirement decisions and may impact cognition post-retirement. Here, we aimed to examine how subclinical depressive symptoms and job stress in working and retired older adults impacted emotional memory. We found that retired, but not working, older adults with greater depressive symptoms showed enhanced negative and impaired positive memory. Second, working older adults with moderately high current job stress showed better memory overall but a weaker positivity bias, while retired older adults with moderately high retrospective job stress showed worse memory overall and a stronger positivity bias. These findings suggest that subclinical depressive symptoms and job stress have differing impacts on emotional memory in late life depending on retirement status.

## Introduction

Retirement is a period of significant change, as older adults transition from the workforce to having unstructured leisure time. Engaging in mental stimulation from one’s occupation can help protect cognition in late life and delay dementia onset^1,2^. When this form of mental stimulation ends, such as in retirement, cognitive performance may begin to deteriorate, in line with the “use it or lose it” hypothesis^1,2^. Supporting this hypothesis, retirement has generally been associated with declines in cognition and increased risk of dementia, beyond typical age-related decline^3–8^. However, there are many individual differences that come with retirement, all of which can significantly impact cognition, mood, and overall well-being. There have been mixed findings regarding the relationship between retirement and depression, in which some studies have found that retirement is associated with declines in depressive symptoms^9^, while others have found that depressive symptoms increase after retirement^10^. Clinical diagnosis of major depressive disorder (MDD) must meet all criteria outlined in the Diagnostic Statistical Manual-5 (DSM-5), in which one must experience five or more depressive symptoms (depressed mood, loss of pleasure, sleep disturbances, etc.) that persist for two or more weeks in a row^11^. Subclinical depression, on the other hand, refers to the presence of meaningful depressive symptoms without meeting the full diagnostic criteria for MDD (e.g. less than 5 depressive symptoms)^12^. Experiencing subclinical depressive symptoms is common in late life, with an estimated prevalence around 13%^13,14^. Subclinical depressive symptoms can not only significantly increase medical illness burden^15^ and risk of developing major depression^16^, but can also impact rates of cognitive decline, where each one-point increase on depression questionnaires, such as the Geriatric Depression Scale (GDS)^17^, significantly increases dementia risk^18–20^.

These differing trajectories of depressive symptoms in retired adults could be due to the change in daily activities of the workplace environment^21^. High levels of job stress have been associated with impaired memory^22,23^ and greater cognitive decline^23–25^, as well as an increased risk of developing depression and dementia^26^. For example, Andel and colleagues assessed 3,779 participants from the Health and Retirement Study (HRS), a longitudinal cohort that began in 1992^23^. They found that those with higher job strain, a measure that incorporates levels of job demands relative to levels of job control, had both worse memory before retirement as well as faster rates of memory decline after retirement^23^. The accumulated stress may negatively impact hippocampal-dependent memory and cognitive health^23^. A recent study by Nilsen et al. that examined 307 participants from the Swedish Adoption/Twin Study of Aging (SATSA), which followed pairs of twins over the course of 27 years^22^, found that those with higher levels of lifetime job strain had worse memory pre-retirement. However, in contrast to Andel et al., they found that higher job strain was associated with slower rates of cognitive decline post-retirement^22^. Thus, retirement may offer a reprieve from work-related stress and improve cognition and well-being^22^. Alternatively, moderate levels of stress at work may keep people feeling challenged and engaged, as well as promoting job satisfaction^27,28^. While these alternatives appear to be in opposition with one another, they may follow principles of the Yerkes-Dodson law, such that moderate levels of stress are beneficial for cognition while too high or too low levels of stress may be detrimental^29,30^. Thus, job stress may confer both protective and harmful effects on cognitive function.

In particular, episodic memory, or memory for events^31–33^, often declines with age and depression^34–36^. However, not all aspects of episodic memory are impacted in the same way. Emotional memory is particularly sensitive to changes in mood, stress, and aging^36–38^. There is evidence of a negativity bias in memory in depression, where memory for negative relative to neutral or positive stimuli is enhanced^36,39–41^. In contrast, a positivity bias in memory has often been reported in aging^37,42–44^. One framework of episodic memory that has been shown to be more sensitive towards capturing age-related memory decline, as well as emotional memory biases across clinical conditions, compared to traditional memory tasks is a pattern separation framework^45^. Hippocampal pattern separation is a computation that reduces interference across experiences with overlapping content, allowing us to remember distinct events^46,47^. Aging, stress, and depressive symptoms have been shown to be associated with poor hippocampal pattern separation across species^36,40,45,48^, as well as the emotional memory biases mentioned above^40,49–52^. Mnemonic discrimination tasks have been developed to tax hippocampal pattern separation through the inclusion of stimuli that are similar but not identical to those seen during encoding, i.e., “lures”, thereby increasing interference in memory^48^. Emotional mnemonic discrimination tasks are also sensitive to subtle changes in emotional memory, making them ideal tests of emotional memory biases^52–55^. Stress and depression have been associated with increased negative mnemonic discrimination ^40,49,51,52^, which relies on hippocampal pattern separation, while aging has been associated with worse mnemonic discrimination and a positivity bias in memory^42–44,50^. Recently, we reported that the positivity bias in aging, as measured through an emotional mnemonic discrimination task, was selective to retired relative to working older adults^56^. However, it is unclear how individual differences, such as subclinical depressive symptoms and job stress, may interact with retirement status to impact the presence of these emotional memory biases. The opposing emotional biases (i.e., negativity bias in stress and depression^36,39,40,57^ and positivity bias in aging^42–44^ and retirement^56^) may influence memory performance in distinct ways.

In the current study, we aimed to examine whether retirement status interacts with subclinical depressive symptoms and job stress to impact emotional memory through the use of an emotional mnemonic discrimination task^52^. We recruited cognitively normal retired and working older adults who were matched across age, sex, and education who were part of a larger study designed to assess retirement impacts on cognition, recently reported in Ferguson et al.^56^. In regard to subclinical depressive symptoms, we hypothesized that greater subclinical depressive symptoms would be associated with impaired general memory, impaired positive memory, and enhanced negative memory in both working and retired older adults, with greater declines in retired older adults due to the additional burden of post-retirement cognitive decline. Additionally, we predicted these effects would be greater for lure discrimination compared to target recognition measures given the sensitivity of each measure to age-related memory impairment. In regard to job stress, we hypothesized that moderate levels of job stress while working may be associated with enhanced cognition (i.e., some stress may be beneficial for maintaining cognition), and one would lose these benefits upon retirement. Additionally, while too much or too little levels of stress while working may be associated with worse cognition, retirement may be associated with enhanced cognition (i.e., removing harmful effects of stress). We also predicted a shift in emotional memory biases, such that retiring from a stressful job may result in a stronger positivity bias (and reduced negativity bias) while currently working a stressful job may result in a weaker positivity bias (and a stronger negativity bias).

## Methods

### Data availability, transparency, and openness

All raw, de-identified data and materials included in the manuscript can be found at GitHub: https://github.com/lealmemorylab/retirement-mental-health. Analyses were conducted in SPSS Statistics Version 28.0.1.1^58^ and R Studio^59^. We report all measures and provide justification of our sample size using power sensitivity analyses. This study’s design, hypotheses, and analysis plan were not pre-registered.

### Participants

The study consisted of working and retired older adults (age range 50–83, N = 86). Inclusion criteria consisted of being age 50 or older, having no diagnosed major psychiatric or medical illness, and having access to a computer with stable internet connectivity. All participants were cognitively healthy (i.e., scored a 24 or above on the MMSE)^60^. Participants were categorized into those who were currently working (N = 35) and those who reported they were retired (N = 51), in which 14 retired older adults were engaged in part-time work (range of 3–10 h per week). Demographic information (i.e., race, sex, education) was collected as part of a demographics questionnaire developed in the lab. Ethnicity was characterized as “Hispanic” or “Non-Hispanic”. Race was self-identified as Black, Asian, Native Hawaiian/Pacific Islander, Arab/Middle Eastern/North African, White, American Indian/Alaska Native/Indigenous, and/or Other. Sex was self-identified as female, male, transgender, nonbinary, prefer not to say, and/or other. Lastly, education was self-assessed as number of years of education (i.e., high school = 12, bachelor’s degree = 16, etc.).

The study was conducted via Zoom, as the study took place during the COVID-19 pandemic. Participants were required to have access to a computer with a camera and a stable internet connection, and to be in a quiet room for the duration of the study. Informed consent was obtained from all participants, and the study procedures were approved the Rice University Institutional Review Board (Study title: Cognitive, MRI, and PET studies of memory systems across the lifespan; Name of institution granting approval: Rice University; Protocol number: IRB-FY2020-6) and carried out in accordance with the relevant guidelines and regulations. Table 1

**Table 1:** Demographics of working and retired older adult participants.

Variable	Working Mean (*SD*)	Retired Mean (*SD*)
N	35	51
Age	63.7 (*8.16*)	66.2 (*6.33*)
Sex	30F	36F
Education (years)	16.5 (*2.50*)	16.8 (*2.38*)
American Indian/Alaska Native	0 (0%)	1 (0.01%)
Asian	2 (0.05%)	3 (0.05%)
Black	3 (0.08%)	4 (0.07%)
Hispanic	2 (0.05%)	9 (17.6%)
Native Hawaiian/Pacific Islander	0 (0%)	1 (0.01%)
White	28 (80%)	32 (62.7%)
Multi-racial/Multi-ethnic	0 (0%)	1 (0.01%)
Other	0 (0%)	0 (0%)
***Neuropsychological Tests***		
MMSE*	29.30 (*0.71*)	28.80 (*1.14*)
RAVLT – Immediate	10.57 (*3.19*)	10.33 (*3.02*)
RAVLT – Delay	10.08 (*2.85*)	9.74 (*3.36*)
Digit Span – Forward	10.71 (*2.45*)	10.13 (*2.72)*
Digit Span – Backward	7.91 (*2.07*)	7.47 (*2.27*)
Letter Number Sequencing	19.20 (*3.68*)	17.74 (*4.59*)
GDS	1.57 (*1.68*)	1.64 (*1.38*)
***Occupational Measures***		
Job Stress	2.51 (*0.74*)	2.74 (*0.86*)
Income	6.6 (*1.75*)	6.03 (*1.88*)

**Key: MMSE:** Mini Mental State Exam; **RAVLT:** Rey Auditory Verbal Learning Test; **GDS:** Geriatric Depression Inventory. **Job Stress:** Range 1–4. **Income:** Range 1–8 (see Supplementary Information 2). *Asterisk indicates a significant difference between groups

### Emotional mnemonic discrimination task

**Fig. 1:**
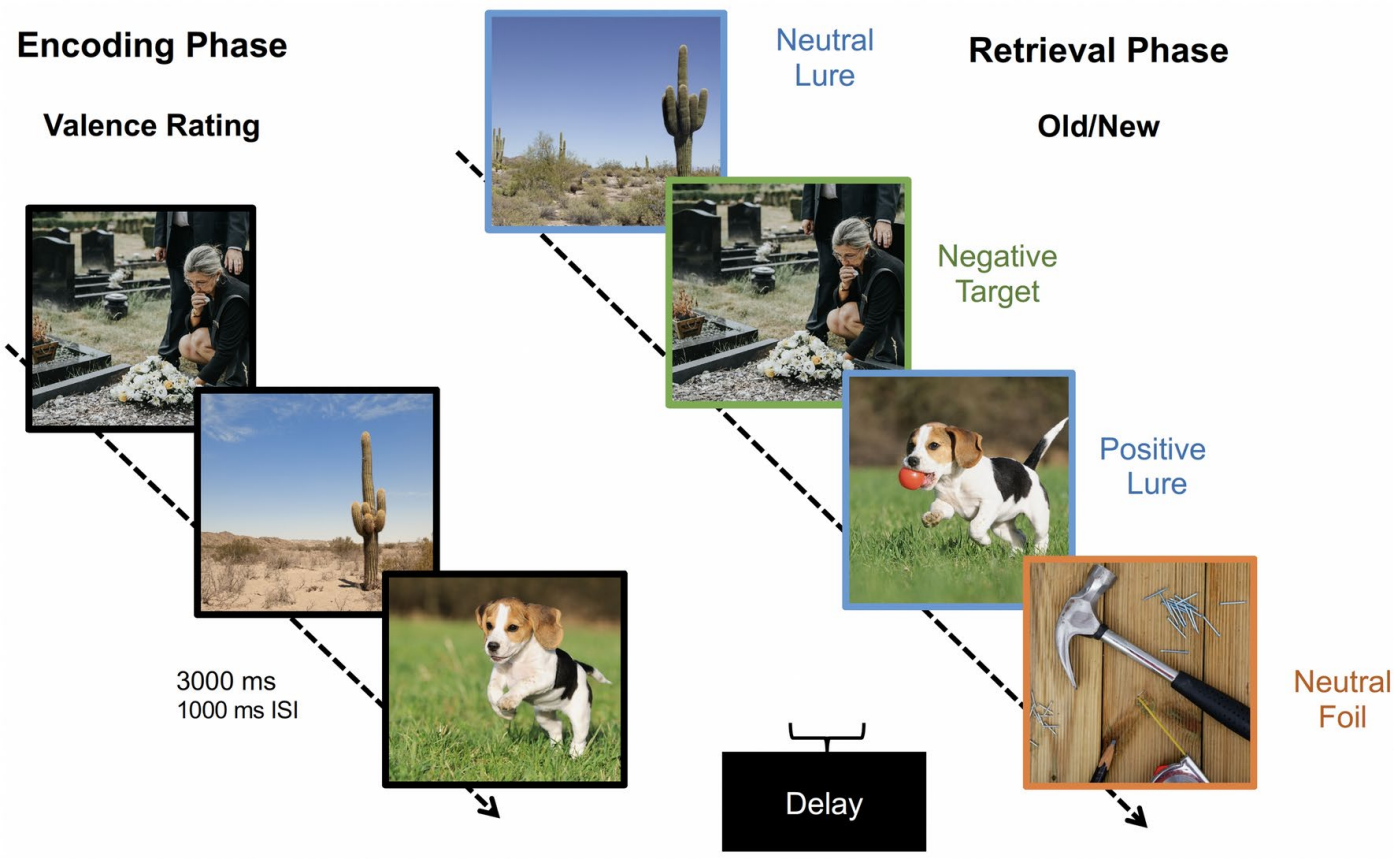
Emotional mnemonic discrimination task. During the Encoding Phase, participants were shown a series of scenes and were asked to rate the emotional content as “negative”, “neutral”, or “positive”. During the Retrieval Phase, images were either repeated (target), similar but not identical (lure), or new images (foil) compared to those shown during encoding. Images licensed from Shutterstock.

Participants completed a well-validated emotional mnemonic discrimination task (Fig. 1) that includes images of positive, negative, and neutral scenes^52^. The task has been shown to be sensitive to age-related changes in emotional memory^54^ and underlying medial temporal lobe (MTL) function^61,62^. All task stimuli have been rated and balanced for emotional valence and similarity across stimuli^52^. Participants first underwent an encoding phase where they were shown scenes that depicted everyday experiences, such as a family gathering or a person driving. A total of 149 images were presented on the screen for 3000 ms with a 1000 ms inter-stimulus interval (ISI), for a total encoding session time of approximately 10 min. Participants were instructed to rate each image as negative, neutral, or positive. After a 15-min delay, during which participants completed a Retirement Questionnaire (see Supplementary Information 1) and the Lifespan Cognitive Activity Questionnaire^63^, participants were given a surprise memory task.

During the retrieval phase, participants were shown scenes they had previously seen during encoding (targets), similar but non-identical scenes (lures), and scenes that were completely new (foils). They were asked to determine if the images were exactly the same as ones seen during encoding, or new or different in any way. Images were on the screen for 3000 ms with a 1000 ms ISI. Retrieval was split into two phases, where each retrieval phase consisted of 145 images for a total time of approximately 9.6 min per test. Images were randomized for each participant during both encoding and the two retrieval phases.

This emotional mnemonic discrimination task provides two memory measures of interest: target recognition (d’), a standard memory measure that assesses memory for repeated images (i.e., targets), and lure discrimination index (LDI), which taxes hippocampal pattern separation and provides a performance metric of the ability to accurately discriminate lure stimuli from target stimuli. Target recognition was measured by a discriminability index (d’), which is calculated as *d’* = *z[p(‘Old’|Target)] – z[p(‘Old’|Foil)]* (i.e., the proportion of trials where the participant correctly responds “old” to target stimulus – a hit – minus the proportion of trials where the participant incorrectly responds “old” to foils – a false alarm) and corrects for response bias. Lure discrimination was calculated as *LDI* = *p(‘New’|Lure) – p(‘New’|Target)* (i.e., the proportion of trials where the participant correctly responds “new” to a lure stimulus – a correct rejection – minus the proportion of trials where the participant incorrectly responds “new” to a target stimulus – a miss) and corrects for response bias. We also calculated two emotional modulation measures for both target recognition and lure discrimination in order to assess individual differences in emotional relative to neutral memory: *Negativity bias* = *[(negative – neutral)/(negative* + *neutral)]* and *Positivity bias* = *[(positive – neutral)/(positive* + *neutral)]*.

### Neuropsychological tests

After the retrieval phase, participants completed a neuropsychological battery to assess general cognition and mood. Participants completed the Mini Mental State Exam (MMSE) to measure general cognitive function^64^; the Rey Auditory Verbal Learning Test (RAVLT), a test of verbal memory that includes immediate and delayed testing^65^; the Digit Span Test to assess working memory and attention^66,67^; and the Letter-Number Sequencing subtest of the Wechsler Adult Intelligence Scale, which measures working memory^67^. We also administered the Geriatric Depression Scale (GDS), which asks about common symptoms of depression in late life through yes/no questions for a possible total of 15 points^17^. Results are shown in Table 1.

### Questionnaires

Participants also completed a battery of questionnaires to measure a variety of occupational factors, participation in cognitive activities, lifestyle habits, and affective factors (see Supplementary Information 2 for full list and description of questionnaires). These additional questionnaires were not the focus of the current study, but could be important for future research. Of interest to the current study was a measure of job stress, which was assessed through a multiple-choice question on the Retirement Questionnaire (Supplementary Information 1), where 1 indicated “not at all stressful” (N = 6), 2 indicated “mildly stressful” (N = 31), 3 indicated “moderately stressful” (N = 36), and 4 indicated “very stressful” (N = 13). Those who were retired answered retrospectively based on their levels of job stress in their last job before retirement while working older adults answered based on their current levels of job stress.

### Statistical analyses

Analyses were conducted using SPSS Statistics Version 28.0.1.1 and R Studio. Planned comparisons were conducted using repeated-measures ANOVAs and t-tests (two-tailed). Post-hoc contrasts were conducted using Scheffé’s method, a common post hoc contrast used for ANOVAs with more than two levels within a factor to test complex linear and non-linear interactions^68^. Pearson correlations and regressions were conducted when appropriate. Moderations were conducted using the “lm” function in R Studio. The function utilizes hierarchical regression to first test the effect of the predictor variable on the outcome variable, then tests the effect of the moderator variable on the outcome variables, and finally tests the interaction of the predictor variable and the moderator variable on the outcome variable. Significant moderations were then analyzed using the PROCESS module in SPSS to examine Johnson-Neyman intervals, which calculates the values of the moderator along a continuum to determine the point at which the moderator begins influencing the outcome variable^69^. Effect sizes (η_p_^2^ and Cohen’s d) are reported when relevant. Statistical values were considered significant at a final corrected alpha level of 0.05, which controlled for Type I error. Repeated-measures tests were corrected for error non-sphericity using Greenhouse–Geisser correction.

We conducted post-hoc G*Power 3.1.9.7^70^ power sensitivity analyses with an α = 0.05 and power of 0.80 revealed that a sample size of 86 (Retired older adults (OA): N = 51, Working OA: N = 35) could detect a Cohen’s d up to 0.62 for two-tailed t-tests assessing the difference between two independent means (medium effect sizes)^71^. For a sample size of 86, within-factor repeated-measure ANOVAs could detect an F effect size of up to 0.14 (small to medium effect sizes), while between-factor repeated-measure ANOVAs would detect an F effect size of up to 0.26 (medium effect size)^71^. Within-between interactions for repeated-measure ANOVAs could detect an F effect size of up to 0.12 (small to medium effect sizes)^71^. For a sample size of 35 (working OAs), within-factor repeated-measure ANOVAs with three measurements could detect an F effect size of up to 0.21 (small to medium effect sizes), while between-factor repeated-measure ANOVAs with two groups would detect an F effect size of up to 0.39 (medium to large effect sizes)^71^. Within-between interactions for repeated-measure ANOVAs could detect an F effect size of up to 0.21 (small to medium effect sizes)^71^. For a sample size of 51 (retired OAs), within-factor repeated-measure ANOVAs with three measurements could detect an F effect size of up to 0.18 (small effect sizes), while between-factor repeated-measure ANOVAs with two groups would detect an F effect size of up to 0.32 (medium to large effect sizes)^71^. Within-between interactions for repeated-measure ANOVAs could detect an F effect size of up to 0.18 (small effect sizes)^71^.

## Results

### No differences in age, sex, or education across working and retired groups

First, we confirmed that there were no statistically significant differences in key demographic features between our working (N = 35) and retired (N = 51) groups. There were no significant differences across age [*t*(84) = 1.52, *p* = 0.114, *d* = 0.35], sex [*t*(84) = 1.92, *p* = 0.058, *d* = 0.40], and education (average of 16 years) [*t*(84) = 0.50, *p* = 0.616, *d* = 0.11]. While the effect of sex was non-significant, there were marginally more female participants in the working OA group relative to the retired OA group. Participants were confirmed to be cognitively normal as characterized by the MMSE (all above a score of 26), where a score of 24 is the threshold for cognitive impairment^64^. Although the working and retired OA groups had comparable performance on all other neuropsychological tests (see Table 1), retired OA showed worse performance on the MMSE compared to their working peers [*t*(84) = 2.53,* p* = 0.013, *d* = 0.51].

### Subclinical depressive symptoms associated with better memory for negative stimuli in retirement

We aimed to examine how subclinical depressive symptoms as measured by the GDS, assessed both at a group-level (high and low) and continuously, impacted performance on an emotional mnemonic discrimination task in working and retired older adults. Most participants in the study exhibited subclinical depressive symptoms on the GDS, or below the 5-point threshold indicative of depression (range of 0 – 9 out of a possible score of 15)^72,73^. We predicted that higher levels of depressive symptoms would be associated with impaired lure discrimination relative to target recognition, particularly in the retired group, a weakened positivity bias, and a stronger negativity bias. Given the limited range of responses on the GDS, we split participants into two groups (high and low GDS). We utilized k-means clustering^74^ to cluster underlying data into groups based on the nearest mean. The elbow method^75^ suggested that 3 groups were the appropriate number of clusters when examining the range of GDS scores: low GDS (GDS range: 0–1; working: N = 21, retired: N = 27), moderate GDS (GDS range: 2–4; working: N = 13, working: N = 23) and high GDS (GDS range: 6–9; working: N = 1, retired: N = 1). Given the sample size of the high GDS cluster, we combined the moderate and high GDS clusters: low GDS (GDS range: 0–1; working: N = 21, retired: N = 27) and high GDS (GDS range: 2–9; working: N = 14, working: N = 24). There were no significant differences in the overall levels of depressive symptoms across working and retired groups [*t*(84) = -0.22, *p* = 0.820, *d* = 0.05].

We conducted a mixed-method ANOVA with emotion (negative, neutral, positive) as the within-subjects factor and GDS scores (high and low) as the between-subjects factor, with separate models for both working and retired older adults and for each memory measure. When examining target recognition as the outcome measure, working older adults showed a main effect of emotion [*F*(2, 66) = 4.79, *p* = 0.012, *η*_*p*_^*2*^ = 0.127], where neutral stimuli were better remembered than emotional stimuli [*F*(1, 33) = 6.27, *p* = 0.017, *η*_*p*_^*2*^ = 0.160]. However, there was no effect of GDS [*F*(1, 33) = 1.52, *p* = 0.226, *η*_*p*_^*2*^ = 0.044] and no interaction between emotion and GDS [*F*(2, 66) = 1.58, *p* = 0.213, *η*_*p*_^*2*^ = 0.046] (Supplementary Fig. 1A). Retired older adults showed a main effect of emotion [*F*(2, 98) = 3.99, *p* = 0.022, *η*_*p*_^2^ = 0.075], where neutral stimuli were remembered better than emotional stimuli [*F*(1, 49) = 4.87,* p* = 0.032, *η*_*p*_^*2*^ = 0.090], and a significant interaction between emotion and GDS group [*F*(2, 98) = 3.12, *p* = 0.049, *η*_*p*_^*2*^ = 0.060], such that those with higher depressive symptoms showed better memory for negative stimuli [*F*(1, 49) = 6.69, *p* = 0.013, *η*_*p*_^*2*^ = 0.120]. There was no effect of GDS overall [*F*(1, 49) = 0.16, *p* = 0.689, *η*_*p*_^*2*^ = 0.003] (Supplementary Fig. 1B).

**Fig. 2:**
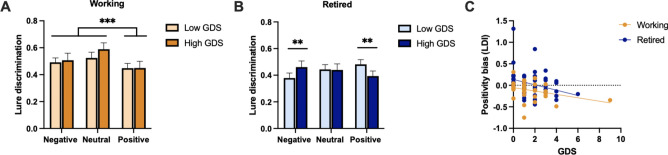
Relationship between depressive symptoms and emotional memory in working and retired older adults. **A**) Lure discrimination performance in working older adults with high and low GDS scores. **B**) Lure discrimination performance in retired older adults with high and low GDS scores. **C**) Correlation between GDS scores and positivity bias in working and retired older adults. Error bars are standard error of the mean (SEM). Asterisks indicate statistical significance (see results for specific main effects and interactions).

When assessing lure discrimination as the outcome variable, working older adults showed a main effect of emotion [*F*(2, 66) = 5.35, *p* = 0.008, *η*_*p*_^*2*^ = 0.140], where neutral stimuli were better remembered than emotional stimuli [*F*(1, 33) = 11.48,* p* = 0.002, *η*_*p*_^*2*^ = 0.258], but there was no effect of GDS [*F*(1, 33) = 0.31, *p* = 0.577, *η*_*p*_^*2*^ = 0.010] and no interaction between emotion and GDS [*F*(2, 66) = 0.51, *p* = 0.588, *η*_*p*_^*2*^ = 0.015] (Fig. 2A). Retired older adults showed no main effect of emotion [*F*(2, 98) = 0.45, *p* = 0.629, *η*_*p*_^*2*^ = 0.009] and no effect of GDS [*F*(1, 49) = 0.005, *p* = 0.942, *η*_*p*_^*2*^ = 0.000]. However, there was a significant interaction between emotion and GDS [*F*(2, 98) = 6.16, *p* = 0.003, *η*_*p*_^*2*^ = 0.112], such that retired older adults with higher depressive symptoms showed worse positive lure discrimination and enhanced negative lure discrimination compared to those with lower depressive symptoms [*F*(1, 49) = 10.94, *p* = 0.002, *η*_*p*_^*2*^ = 0.183] (Fig. 2B).

We also assessed depressive symptoms continuously in order capture variability of depressive symptom severity. We examined negativity and positivity bias measures to determine how depressive symptoms impacted emotional memory biases. Correlation analyses revealed a significant negative association between GDS and LDI positivity bias scores in retired OA [*r*(49) = -0.29, *p* = 0.037] and a marginal negative association in working OA [*r*(33) = -0.31, *p* = 0.065] (Fig. 2C). There were no associations between GDS and target recognition positivity bias in either retired [*r*(51) = -0.19, *p* = 0.190] or working [*r*(35) = -0.10,* p* = 0.557] older adults. When examining correlations with negativity bias scores, there were no significant correlations between GDS and LDI negativity biases in either working [*r*(33) = 0.25, *p* = 0.134] or retired older adults [*r*(49) = 0.08,* p* = 0.562], no relationship between GDS and target recognition negativity bias in the working group [*r*(35) = 0.22, *p* = 0.207], and a marginal positive association between GDS and target recognition negativity bias in the retired group [*r*(51) = 0.26, *p* = 0.060].

### Opposing effects of job stress on lure discrimination in working and retired older adults

Next, we aimed to examine how job stress, either current job stress in working OA or retrospective job stress in retired OA, may impact emotional memory. We hypothesized that moderate levels of job stress while working may be associated with enhanced cognition (i.e., some stress may be beneficial for maintaining cognition), in which one would lose these benefits upon retirement, while too much or too little levels of stress while working may be associated with worse cognition, in which retirement may be associated with enhanced cognition (i.e., removing harmful effects of stress). We also predicted a shift in emotional memory biases, such that retiring from a stressful job may result in a stronger positivity bias (and reduced negativity bias) while currently working stressful job may result in a weaker positivity bias (and a stronger negativity bias).

We examined job stress both at the group-level and continuously. First, we categorized participants’ responses to the job stress scale into “low” (little to no job stress) and “high” (moderate to high job stress) groups: working (low job stress: N = 16, high job stress: N = 19) and retired (low job stress: N = 21, high job stress: N = 30). K-means clustering and the elbow method confirmed that these two groups represented the best clustering of the data. Overall, there were no significant differences in reported levels of job stress across working and retired older adults [*t*(84) = 1.28, *p* = 0.203, *d* = 0.28].

We conducted a mixed-method ANOVA with emotion (negative, neutral, positive) as the within-subjects factor and job stress (high, low) as the between-subjects factor in both working OA and in retired OA for each memory measure. We first tested models with target recognition as our outcome variable. In working OA we found a main effect of emotion [*F*(2, 66) = 4.14,* p* = 0.021, *η*_*p*_^*2*^ = 0.112], where neutral stimuli were remembered better than emotional stimuli [*F*(1, 33) = 4.33, *p* = 0.045, *η*_*p*_^*2*^ = 0.116]. There was no effect of current job stress [*F*(1, 33) = 0.16, *p* = 0.686, *η*_*p*_^*2*^ = 0.005] and no interaction between emotion and current job stress [F(2, 66) = 0.24, p = 0.780, *η*_*p*_^*2*^ = 0.007] (Supplementary Fig. 2A). Similarly, in retired OA, we found a main effect of emotion [*F*(2, 98) = 3.39, *p* = 0.038, *η*_*p*_^*2*^ = 0.065], where neutral stimuli were remembered better than emotional stimuli [*F*(1, 49) = 4.58, *p* = 0.037, *η*_*p*_^*2*^ = 0.086], but no effect of retrospective job stress [*F*(1, 49) = 0.05,* p* = 0.817, *η*_*p*_^*2*^ = 0.001] and no interaction between emotion and retrospective job stress [*F*(2, 98) = 0.10, *p* = 0.898, *η*_*p*_^*2*^ = 0.002] (Supplementary Fig. 2B).

**Fig. 3:**
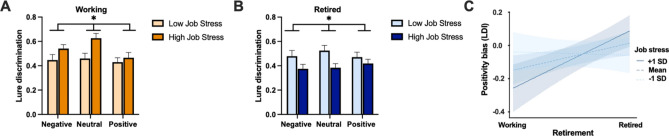
Relationship between job stress and emotional memory in retired and working older adults. **A**) Lure discrimination performance in retired older adults with high and low levels of job stress. **B**) Lure discrimination performance in working older adults with high and low levels of job stress. **C**) Job stress as a moderator of retirement status and positivity bias. Error bars are SEM. Asterisks indicate statistical significance (see results for specific main effects and interactions).

We next assessed models with lure discrimination as the outcome measure. In working OA, we found a significant main effect of emotion [*F*(2, 66) = 4.58, *p* = 0.016, *η*_*p*_^*2*^ = 0.122], where neutral stimuli were remembered better than emotional stimuli [*F*(1, 33) = 10.14, *p* = 0.003, *η*_*p*_^*2*^ = 0.235]. We also found a significant effect of current job stress [*F*(1, 33) = 5.07, *p* = 0.031, *η*_*p*_^*2*^ = 0.133], where those with higher levels of current job stress showed better lure discrimination than those with lower levels of current job stress. However, there was no interaction between emotion and current job stress [*F*(2, 66) = 2.15, *p* = 0.129, *η*_*p*_^*2*^ = 0.061] (Fig. 3A). In retired OA, we found the opposite to be true, with a significant effect of retrospective job stress [*F*(1, 49) = 4.39, *p* = 0.041, *η*_*p*_^*2*^ = 0.082], where those with higher levels of retrospective job stress showed worse lure discrimination than those with lower levels of retrospective job stress. We found no effect of emotion [*F*(2, 98) = 0.59, *p* = 0.542, *η*_*p*_^*2*^ = 0.012] or interaction between emotion and retrospective job stress [*F*(2, 98) = 1.54,* p* = 0.220, *η*_*p*_^*2*^ = 0.031] (Fig. 3B).

Given these findings, we wanted to determine whether job stress moderated the relationship between retirement status and lure discrimination, our outcome measure of interest. We found that there was a marginal interaction between job stress and retirement status, such that higher levels of current job stress were associated with better memory in working OA, whereas lower levels of retrospective job stress were associated with better lure discrimination in retired OA [*β* = -0.78, *t*(82) = -1.88, *p* = 0.063, *R*^*2*^*adj* = 0.043]. A Johnson-Neyman analysis revealed that a job stress level of 2.74 (equivalent to a response of mildly to moderately stressful) was when job stress began to significantly impact lure discrimination performance.

Next, we assessed whether current or retrospective job stress was associated with a positivity or negativity bias in our two groups of interest. We found a significant negative correlation between current job stress and the positivity bias in LDI in working OA [*r*(35) = -0.461, *p* = 0.005], but no such correlation between retrospective job stress and the positivity bias in LDI in retired OA [*r*(51) = 0.256, *p* = 0.070]. There were no significant relationships between target recognition positivity biases in either group, nor between either of the negativity bias measures and job stress measures in either group. We next examined whether job stress moderated the relationship between retirement status and our outcome measure LDI positivity bias. We found a significant interaction between job stress and retirement status, where higher levels of retrospective job stress was associated with a stronger positivity bias in retired OA, but current job stress was associated with a weaker positivity bias in working OA [*β* = 1.20, *t*(82) = 3.05, *p* = 0.003, *R*^*2*^*adj* = 0.138] (Fig. 3C). A Johnson-Neyman analysis revealed that job stress levels of 2.44 (mildly to moderately stressful) is when job stress becomes significantly predictive of positivity bias in LDI.

Finally, we wanted to determine which factors described in the current study were most strongly predictive of the positivity bias in LDI. We conducted a stepwise linear regression including GDS, job stress, retirement status, age, sex, and education as variables. We found that GDS was the strongest predictor of the positivity bias in LDI [*β* = -0.27, *t*(1, 84) = 2.60, *p* = 0.011, *R*^*2*^*adj* = 0.064]. A second model added retirement as a predictor [Δ* R*^*2*^*adj* = 0.077]. Thus, even subclinical depressive symptoms strongly influence the presence of a positivity bias in aging, beyond the effects of current or retrospective job stress.

## Discussion

In the current study, we investigated how subclinical depressive symptoms and current and retrospective job stress impacted memory in working and retired older adults using an emotional mnemonic discrimination task. We found that retired older adults with subclinical depressive symptoms showed enhanced negative lure discrimination, but worse positive lure discrimination. When we examined emotional memory bias (i.e., memory for positive/negative relative to neutral stimuli), we found that greater depressive symptom severity was associated with a lower positivity bias in lure discrimination only in retired older adults. More succinctly, these findings indicate that retired older adults, but not working older adults, with more depressive symptoms have better memory for negative stimuli and worse memory for positive stimuli. These results are consistent with a memory bias towards negative stimuli^39,57^ and away from positive stimuli^39,76,77^ commonly reported in depression. Depressive symptoms in late life can therefore reduce the positivity bias commonly reported in the aging literature, which may be exacerbated by retirement^42–44^. We extend this finding to show that even subclinical levels of depressive symptoms can reduce the positivity bias in memory reported in aging. This suggests that the positivity bias in aging is not universal, and that even subtle individual differences can impact its presentation. Interestingly, we did not find the effect of subclinical depressive symptoms on negative and positive lure discrimination in working older adults, suggesting that continuing to work may help protect against the deleterious impacts of subclinical depression. This may be partly explained by social factors. Loneliness^78^ and low levels of social support^79^ are significant risk factors for the development of depression and often increase with retirement^80–84^. However, social interactions and social support received at work can counteract loneliness and help protect against the detrimental effects of depression^85,86^. Social support may buffer the effects of the negativity bias and reduce negative affect^87–89^. Thus, workplace social support may help reduce the negativity bias in working older adults with subclinical depression. However, this moderating effect of social support will need to be explored directly in future studies.

We next examined whether current or retrospective job stress could underlie variability in emotional memory outcomes in working and retired older adults. We found opposing effects of job stress on lure discrimination in working and retired older adults, where working older adults with moderately high levels of current job stress showed better lure discrimination performance, while retired older adults with higher levels of retrospective job stress exhibited worse lure discrimination. Thus, there is a reversal in the impacts of job stress on memory depending on whether a participant is currently working or retired. This aligns with previous research finding job stress is not associated with cognitive decline while working, but became associated with increased cognitive decline after retirement^23^. Jobs with moderate levels of stress and higher job demands may keep workers feeling challenged^27,28^ and boost cognitive performance^90^. This follows principles of the Yerkes-Dodson law, such that moderate levels of stress are associated with optimal cognitive performance^29,30^. Stress levels that are too low do not elicit attentional engagement or interest, whereas stress levels that are too high subsume cognitive resources and impair task performance^29,30^. Here, our results appear to be consistent with stress levels in the moderate range, such that moderate stress while working may benefit cognitive performance, and once these benefits are removed in retirement, this may negatively impact memory. The challenge of working jobs with more mental demands may help maintain cognitive abilities, in line with the “use it or lose it” hypothesis^1,2^, and also stimulate release of stress hormones (e.g., epinephrine) that may boost lure discrimination performance^49^. Lure discrimination measures may be especially sensitive to variations in stress^45,49,91^, as we did not find these same effects for target recognition. The dentate gyrus (DG) and CA3 hippocampal subfields that support pattern separation computations are vulnerable to stress hormones, where chronic exposure to high levels of stress may impair DG/CA3 function and reduce pattern separation abilities^36,92,93^. In contrast, moderate levels of stress may enhance pattern separation abilities^49^.

We also assessed how current and retrospective job stress impacted the presence of the positivity bias in memory. We found that higher levels of current job stress in working older adults was associated with a weaker positivity bias in lure discrimination, while higher levels of retrospective job stress were associated with a stronger positivity bias in lure discrimination in retired older adults. Interestingly, this also indicates inverse impacts of job stress on memory for positive stimuli depending on one’s retirement status. Higher levels of current stress have been associated with impaired memory for positive and neutral stimuli^49,94,95^, as well as improved memory for negative stimuli^49,96^. Additionally, higher job stress can impact well-being and has been associated with increased rates of depression while in the stressful occupational environment^97–100^. However, retiring from stressful jobs may offer a reprieve from such environments and enhance well-being ^22,101^. This improved well-being may lead to enhanced focus on positive events and experiences and thus a stronger positivity bias in memory^42,43^. However, longitudinal studies will be needed in order to determine how job characteristics impact well-being and emotional memory both before and after retirement.

The positivity bias is not universal in late life. We have demonstrated that a variety of factors, including retirement status, depressive symptoms, and job characteristics, moderate the strength of this bias. Thus, taking a broad range of experiences into account is essential for gaining a deeper understanding of how and why a positivity bias in memory may manifest and in whom. In the current study, we found that depressive symptoms and retirement status were the strongest predictors of the positivity bias in memory in aging. This suggests that there are core vulnerabilities that underlie emotional memory biases. Policies focused on supporting mental health before and after retirement could impact the emotional memory biases we report here and could potentially improve cognition, well-being, and reduce risk of dementia. Workplace policies promoting social support could help mitigate the harmful effects of depressive symptoms and job stress. Programs aimed at developing social support networks for retired older adults would also be essential for reducing depression and improving quality of life. Additionally, exploring individual differences such as social support, workplace support, loneliness, and cognitive stimulation both in and outside of the workplace in future studies will be essential for gaining a more nuanced understanding of how memory and the positivity bias in memory manifest in aging. For instance, future studies with a greater focus on social support in and out of the workplace could further home in on if there are 1) instrumental differences in social support received in the workplace versus in retirement, and 2) what aspects of social support, such as family support or the quality of social connections, are most beneficial for buffering depressive symptoms.

The current study has several important limitations. First, the sample size is relatively small, and the participants are predominately non-Hispanic White (69.4%), with high levels of both education (average of 16.7 years of education) and income (an average of $80,000 to $100,000). These individual differences are important to consider given that those with lower socioeconomic status and Black and Hispanic adults have fewer occupational opportunities and less access to high skill and high-income careers^102,103^. These groups also have higher depression risk and more severe depressive symptoms^104–106^. Thus, there is a need for a more diverse sample in order to understand how retirement, depressive symptoms, and occupational characteristics impact emotional memory in order to better represent people’s lived experiences.

Another limitation is that the study was conducted in the midst of the COVID-19 pandemic over Zoom. Participants were required to have access to a computer with a camera and microphone, a stable internet connection, and a quiet room for the duration of the study. However, we had less control of their environment in general. This is certainly one of the contributing factors for our lack of socioeconomic diversity. The COVID-19 pandemic may have also contributed to increased levels of stress, depression, and isolation during the time the study was conducted^107,108^. Thus, it will be important for future studies to examine how retirement status, depression, and job stress impact emotional memory in a more diverse sample and during a more representative point in time.

Additionally, the current study was conducted cross-sectionally. Longitudinal studies could better track changes in stress and depression in the workplace, during the retirement transition, and post-retirement. There are many different factors that can influence levels of stress and depression before and after retirement^101,109^. Some retirees may transition out of fulfilling work into a stressful retirement where they are responsible for caregiving for a spouse or grandchildren. In contrast, others may have very stressful jobs that contribute to greater depressive symptoms, and the transition to retirement offers a reprieve from that stress and a chance to reduce depressive symptoms. Thus, longitudinal studies could offer a more nuanced examination of these differing work and retirement trajectories.

In the current study, we examined subclinical depressive symptoms, however, it will be important for future studies to assess how a broader range of depressive symptoms – from non-existent to severe – impact emotional memory. Those with more severe depressive symptoms exhibit stronger negativity biases^40^, greater rates of cognitive decline^34,110^, and increased risk of dementia^34,111–113^. How retirement status may moderate these relationships remains to be explored. Additionally, our measure of job stress was assessed on a scale of 1 to 4, thereby limiting variability of responses. Future studies would benefit from using not only a more robust scale of job stress, but also one that includes different aspects of job stress, such as job demands (i.e., the amount of work to be done) and job control (i.e., the level of autonomy to complete the work)^114^. These dimensions have important impacts on cognition and well-being^22,27,90,99^. Lastly, it will be important for future studies to examine additional information about occupational characteristics that may influence these results. Future studies designed to explore these questions in more depth are warranted. This can provide important nuance when examining how job stress and depressive symptoms impact memory and well-being.

## Supplementary Information

Supplementary Information.
