# Supplementary material for: Subclinical depressive symptoms and job stress differentially impact memory in working and retired older adults
Source: Sci Rep. 2025 Jan 25;15:3163. doi: 10.1038/s41598-025-87333-9 (PMC11763037; doi:10.1038/s41598-025-87333-9)
Supplement: Supplementary file 1 — Supplementary Information. [file 41598_2025_87333_MOESM1_ESM.pdf]

Supplementary Information 1

Retirement Questionnaire

Are you retired? Circle one. Yes No

SECTION I: Education, income, and work history

1. **What is the highest degree you earned?**
   - ☐ High school diploma or equivalency (GED)
   - ☐ Associate degree (junior college)
   - ☐ Bachelor's degree
   - ☐ Master's degree
   - ☐ Doctorate
   - ☐ Professional (MD, JD, DDS, etc.)
   - ☐ Other: ________________
   - ☐ None of the above (less than high school)

2. **In which kind of business or industry did you work (or currently work)?**

   ____

3. **What was your job title before you retired? If you are not retired, what is your job title?**

   ____

4. **How much did you earn, before taxes and other deductions, in your last job before retirement? If you are not retired, what do you currently earn?**
   - ☐ $10,000 through $20,000
   - ☐ $20,000 through $40,000
   - ☐ $40,000 through $60,000
   - ☐ $60,000 through $80,000
   - ☐ $80,000 through $100,000
   - ☐ $100,000 through $150,000
   - ☐ $150,000 and greater

5. **How many people are currently living in your household, including yourself?**
   Number of people: _____
   Of these people, how many are children? _____
   Of these people, how many are adults? _____
   Of the adults, how many bring income into the household? _____
   Any pets? _______ How many pets and what type?____________________________

6. **What is your marital status?**
   - ☐ Single
   - ☐ Married
   - ☐ Divorced
   - ☐ Widowed

7. **What is your primary source of income? Please select all that apply.**
   - ☐ Full-Time Job
   - ☐ Part-Time Job
   - ☐ Retirement Accounts (such as 401(k), IRA, etc.)
   - ☐ Social Security
   - ☐ Stocks
   - ☐ Savings
   - ☐ Pension
   - ☐ Rent & Royalties
   - ☐ Inheritance
   - ☐ Annuities or Insurance
   - ☐ Home Equity
   - ☐ Spouse or Partner Income
   - ☐ Other: ________________

8. **Which of these categories best describes your total combined family income for the past 12 months? This should include income (before taxes) from all sources, wages, rent from properties, social security, disability and/or veteran's benefits, unemployment benefits, workman's compensation, help from relatives (including child payments and alimony), and so on.**
   - ☐ $10,000 through $20,000
   - ☐ $20,000 through $40,000
   - ☐ $40,000 through $60,000
   - ☐ $60,000 through $80,000
   - ☐ $80,000 through $100,000
   - ☐ $100,000 through $150,000
   - ☐ $150,000 and greater

9. **Is the home where you live:**
   - ☐ Owned or being bought by you (or someone in the household)?
   - ☐ Rented for money?
   - ☐ Occupied without payment of money or rent?
   - ☐ Other: ___________________________________

SECTION II: In this section, we will be discussing your activities and social life **BEFORE** retirement or if you are currently working. Please base your answers on the last five years at work before retirement.

1. **How many hours per week did you work at your job (or how many hours do you currently work)?**
   - ☐ 5-10 hours per week
   - ☐ 10-20 hours per week
   - ☐ 20-30 hours per week
   - ☐ 30-40 hours per week
   - ☐ 50-60 hours per week
   - ☐ 60 + hours per week

2. **How would you rate your <u>job satisfaction</u>?**

   Not at all satisfied Mildly satisfied Moderately satisfied
Very satisfied

3. **How would you rate your <u>job stress level</u>?**

   Not at all stressful Mildly stressful Moderately stressful
Very stressful

4. **Before retirement**, was your participation in each of the following activities closest to daily, weekly, monthly, or less often or never? <u>If you are still working</u>, how often are you participating in each of the following activities? Your best guess is fine.

   Family or friendship activities outside the household

   Daily Weekly Monthly Less often or never

   Church or religious activities such as services, committees, or choirs

   Daily Weekly Monthly Less often or never

   Physical activity or exercise with other people

   Daily Weekly Monthly Less often or never

   Other recreational activities involving other people, including hobbies, bingo and other games

   Daily Weekly Monthly Less often or never

   Educational and cultural activities involving other people such as attending courses, concerts or visiting museums

Daily Weekly Monthly Less often or never

Neighborhood, community or professional association activities

Daily Weekly Monthly Less often or never

Volunteer or charity work

Daily Weekly Monthly Less often or never

5. **Before retirement, what kind of exercise did you engage in? If you are currently working, what kind of exercise do you engage in currently?**
   - ☐ Aerobic (such as running, cycling, walking, etc.)
   - ☐ Strength training (such as lifting weights, Pilates, etc.)
   - ☐ Stretching
   - ☐ Balance exercises
   - ☐ Other: ________________

6. **Before retirement, what hobbies did you engage in at home? If you are currently working, what hobbies do you engage in at home? Select all that apply.**
   - ☐ Reading
   - ☐ Watching movies or TV
   - ☐ Cooking
   - ☐ Gardening
   - ☐ Model building
   - ☐ Arts and crafts
   - ☐ Video games
   - ☐ Board and/or card games
   - ☐ Shopping
   - ☐ Playing musical instruments
   - ☐ Other: ___________________________________

7. **How would you rate your overall happiness in <u>the last five years before retirement</u>? If you are currently working, rate your overall happiness in <u>the last five years.</u>**

Very unhappy Somewhat unhappy Moderately happy
Very happy

8. **How much money did you have saved for retirement? If you are currently working, how much money do you have saved for retirement?**
   - ☐ $10,000 through $20,000
   - ☐ $20,000 through $40,000
   - ☐ $40,000 through $60,000
   - ☐ $60,000 through $80,000
   - ☐ $80,000 through $100,000
   - ☐ $100,000 through $150,000
   - ☐ $150,000 and greater

9. **Did you retire because you wanted to, or was it because of some other circumstance? If you are not retired, skip this question.**
   - ☐ I retired because I wanted to
   - ☐ No, I retired because of other circumstances

10. **If you retired because of other circumstances, please explain those circumstances here.**

    ____

<u>SECTION III</u>: In this section, we will be asking about your activities and social life **<u>AFTER</u>** <u>retirement</u>. Please base your answers on your activities since retirement. **If you are not retired, you have completed the questionnaire.**

1. **How long have you been retired?**

   ____

2. **How old were you when you retired?**

   ____

3. **Have you gotten any jobs since you retired from your primary job?**

   Yes No

   If so, please list your job title(s) and how long you worked there:

   ____

   ____

   If so, what were your reasons for getting a job after retiring?

- ☐ Money
- ☐ Boredom
- ☐ Engagement in the community
- ☐ Other: ____

____

4. **After retirement,** is your participation in each of the following activities closest to daily, weekly, monthly, or less often or never? Your best guess is fine.

Family or friendship activities outside the household

Daily Weekly Monthly Less often or never

Church or religious activities such as services, committees, or choirs

Daily Weekly Monthly Less often or never

Physical activity or exercise with other people

Daily Weekly Monthly Less often or never

Other recreational activities, including hobbies, bingo and other games

Daily Weekly Monthly Less often or never

Educational and cultural activities involving other people such as attending courses, concerts or visiting museums

Daily Weekly Monthly Less often or never

Neighborhood, community or professional association activities

Daily Weekly Monthly Less often or never

Volunteer or charity work

Daily Weekly Monthly Less often or never

5. **After retirement, what kind of exercise do you engage in?**
   - ☐ Aerobic (such as running, cycling, walking, etc.)
   - ☐ Strength training (such as lifting weights, Pilates, etc.)
   - ☐ Stretching

☐ Balance exercises
☐ Other: ________________

6. **Have your <u>exercise habits</u> changed since retiring?**

Exercise more since retiring Same as before Exercise less since retiring

7. **After retirement, do you prefer to go out and do new things or do you prefer to stay home?**

☐ Go out and do new things
☐ Stay home

8. **After retirement, what hobbies do you engage in at home? Select all that apply.**
☐ Reading
☐ Watching movies or TV
☐ Cooking
☐ Gardening
☐ Model building
☐ Arts and crafts
☐ Video games
☐ Board and/or card games
☐ Shopping
☐ Playing musical instruments
☐ Other: ___________________________________

9. **Has the <u>number of hobbies</u> you engage in changed since retiring?**

More hobbies now Same Less hobbies now

10. **How would you rate your <u>overall happiness</u> since you retired?**

Very unhappy Somewhat unhappy Moderately happy Very happy

11. **How have you been <u>sleeping</u> since retiring?**

Much better Slightly better Same as before Slightly worse Much worse

12. Do you feel like your <u>memory</u> has changed since you retired?

Much better Slightly better Same as before Slightly worse Much worse

13. At what age did you feel like your memory got <u>worse</u>?

20-30 30-40 40-50 50-60 60-70 70-80 Hasn't gotten worse

14. If there is anything you wish to add about your experience with retirement that has not been covered in this questionnaire, please list it here.

Supplementary Information 2

Neuropsychological tests

Participants completed a neuropsychological battery to assess general cognition after completing the mnemonic discrimination task. They completed the MMSE, a measure of general cognitive function (Folstein, Folstein, & McHugh, 1975); the Rey Auditory Verbal Learning Test (RAVLT), a measure of verbal memory that includes immediate and delayed testing (Lezak, Howieson, Loring, Hannay, & Fischer, 2004); the Digit Span Test, a measure of working memory and attention (Baddeley, 2000; Wechsler, 2008); and the Letter-Number Sequencing subtest of the Wechsler Adult Intelligence Scale, which measures working memory and task switching abilities (Wechsler, 2008).

Questionnaires

Participants were also administered a series of questionnaires examining various aspects of aging including cognitive activity, occupational factors, lifestyle factors, and affective factors. Two questionnaires – the Retirement Questionnaire and the Lifespan Cognitive Activity Questionnaire – were completed between the encoding phase and testing phase of the mnemonic discrimination task. The remaining questionnaires were administered after completion of the mnemonic discrimination task.

Cognitive activity measures: We administered the Lifespan Cognitive Activity Questionnaire (LCAQ), a well-validated measure of engagement in cognitive activities throughout the lifespan (Wilson, Barnes, & Bennett, 2003). The LCAQ measures participation in cognitively stimulating activities, such as playing board games and reading books, on a scale from 1 to 5 across four lifespan periods: childhood (ages 6-12), young adulthood (age 18), middle age (ages 30-40), and late life (current age).

Scores are added together and averaged within each period of life. A higher average score indicates more participation in cognitive activities. We aggregated the scores across childhood, young adulthood, and middle age to create a *past cognitive activity* score, and the measure of *current cognitive activity* was based on the late life score.

We also created a Retirement Questionnaire that assessed cognitive activity levels both before and after retirement. We calculated a current *cognitive activity* measure which included a checklist of cognitively stimulating leisure activities (reading, watching movies or TV, cooking, gardening, model building, arts and crafts, video games, board and/or card games, shopping, playing musical instruments, other) for a possible total of 11 points. We also used the Retirement Questionnaire to generate a measure of *composite activity* levels. These questions asked about frequency of participation (daily, weekly, monthly, or less often or never) in various social, physical, and cognitive activities (i.e., participation in family or friendship activities, physical exercise, and educational or cultural activities), as well as the checklist of hobbies used in our cognitive activity measure, with a total possible score of 37 points for pre-retirement activity levels and 37 points for post-retirement activity levels.

Lifestyle measures: To examine lifestyle factors, we collected the Pittsburgh Sleep Quality Index, which measures hours of sleep and sleep quality (1 = "very bad", 4 = "very good") (Buysse, Reynolds, Monk, Berman, & Kupfer, 1989); the Berkeley Retrospective Sleep Questionnaire, which measures sleep habits throughout the lifespan (Winer et al., 2019); and the Lifestyle & Exercise Questionnaire, which we created in the lab to examine cognitive, social, and physical activity, as well as dietary habits. *Physical activity* was determined based on responses to questions asking about frequency (0 = "never", 5 = "16 or more times a week") of engagement in physical

activities, such as walking or strength training, as well as the average amount of time spent sitting (1 = “12 hours or more”, 5 = “less than 1 hour”) and standing (1 = “less than 1 hour”, 6 = “more than 8 hours”) in a week, for a possible total of 42 points. Dietary habit scores were based on responses to a 5-point scale asking how often in a given week that food/beverage is consumed, where 0 indicated “less often or never” and 4 indicated “more than once a day”. A *dietary composite* was created in order to generate a measure of a healthy diet, where foods such as red meat, desserts, and sugary drinks were reverse scored, and factors such as fish, caffeine, and green vegetables were positively scored. The dietary composite was composed of 11 items, with a possible total of 44 points. A higher score indicated a healthier diet. Finally, we also administered a COVID-19 questionnaire to determine if participants had been diagnosed with or experienced any symptoms of COVID-19.

Occupational measures: *Income* and *retirement savings* were measured through an eight-point multiple choice scale, where 1 indicated “$0 or N/A” and 8 indicated “$150,000 and greater”. *Job stress* was assessed through multiple choice questions, where 1 indicated “not at all stressful” and 4 indicated “very stressful”. *Occupational cognitive ability* was determined by matching job titles written in the retirement questionnaire to occupational categories listed on the Occupational Information Network (O*NET), a database of occupation information developed by the U.S. Department of Labor (Peterson, Mumford, Borman, Jeanneret, & Fleishman, 1999). Occupational titles found in O*NET were then used to determine worker cognitive abilities. Cognitive ability was calculated by totaling the level scores of 21 factors, such as written comprehension, mathematical reasoning, and deductive reasoning, for a given occupation, a calculation similar to those used in previous studies

utilizing O*NET occupational data (Beier, Torres, Fisher, & Wallace, 2019; Forstmeier & Maercker, 2009). *Age of retirement* and *years retired* were entered as numerical values in free response questions. *Jobs post retirement* was a binary “yes/no” question and *retirement reason* was included as either voluntary or involuntary.

Affective measures: We collected the Beck Anxiety Inventory (BAI), which measures anxiety symptoms during the past month on a zero to three scale (0 = “not at all” and 3 = “severely – it bothered me a lot”) for a possible total of 63 points (Beck, Epstein, Brown, & Steer, 1988); the BDI-II, which assesses depressive symptoms during the past two weeks on a zero to three scale (for example, 0 = “I do not feel I am worthless” and 3 = “I feel utterly worthless”) for a possible total of 63 points (Beck, Steer, & Brown, 1996); the Geriatric Depression Scale (GDS), which measures common symptoms of depression in late life through yes/no questions (e.g., “Do you think it is wonderful to be alive right now?”) for a possible total of 15 points (Burke et al., 1991); the Perceived Stress Scale, which asks about feelings of stress both currently (for a possible total of 10 points) and during the past month (for a possible total of 40 points) (Cohen, 1994); the Subjective Memory Complaints Questionnaire, which measures subjective feelings of memory loss through yes/no questions (e.g., “do you think that your memory is worse than 10 years ago?”) for a total of 14 points and which is often associated with depressive symptoms (Brigola et al., 2015; Youn et al., 2009).

Supplementary Information 3

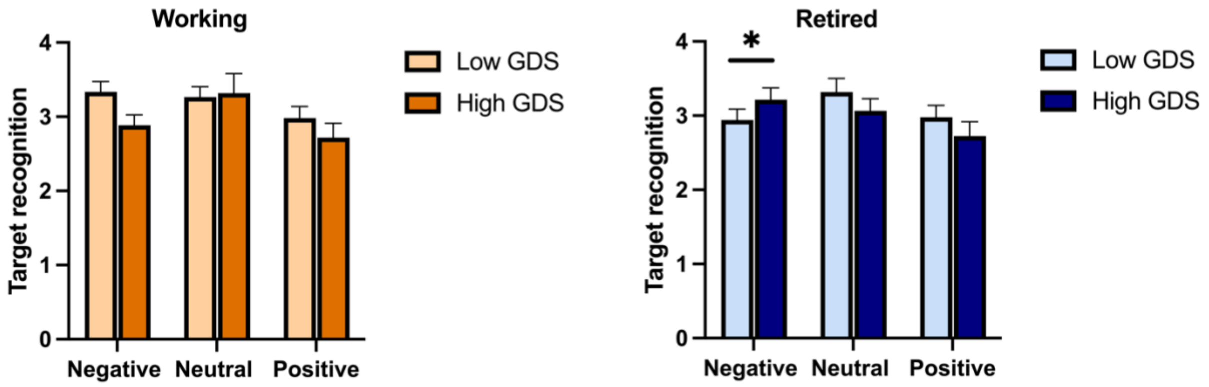

Supplementary Figure 1. Relationship between depressive symptoms and emotional memory in retired and working older adults. A) Target recognition performance in working older adults with high and low GDS scores. A) Target recognition performance in retired older adults with high and low GDS scores. Asterisks indicate statistical significance.

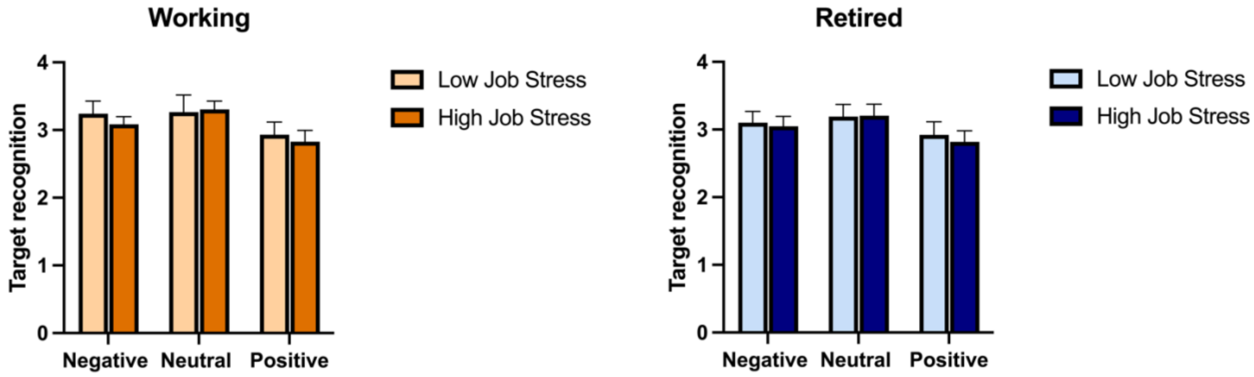

Supplementary Figure 2. Relationship between job stress and emotional memory in retired and working older adults. A) Target recognition performance in working older adults with high and low job stress. A) Target recognition performance in retired older adults with high and low job stress.

Supplementary References

Baddeley, A. (2000). The episodic buffer: a new component of working memory? *Trends in Cognitive Sciences*, *4*(11), 417–423. https://doi.org/10.1016/S1364-6613(00)01538-2

Beck, A. T., Epstein, N., Brown, G., & Steer, R. (1988). Beck Anxiety Inventory . Retrieved 21 July 2022, from https://psycnet.apa.org/record/9999-02025-000?doi=1

Beck, A. T., Steer, R. A., & Brown, G. (1996). Beck Depression Inventory–II. Retrieved 21 July 2022, from https://psycnet.apa.org/record/9999-00742-000?doi=1

Beier, M. E., Torres, W. J., Fisher, G. G., & Wallace, L. E. (2019). Age and job fit: The relationship between demands–ability fit and retirement and health. *Journal of Occupational Health Psychology*, *25*(4), 227. https://doi.org/10.1037/OCP0000164

Brigola, A. G., Manzini, C. S. S., Oliveira, G. B. S., Ottaviani, A. C., Sako, M. P., & Vale, F. A. C. (2015). Subjective memory complaints associated with depression and cognitive impairment in the elderly: A systematic review. *Dementia & Neuropsychologia*, *9*(1), 51. https://doi.org/10.1590/S1980-57642015DN91000009

Burke, W. J., Roccaforte, W. H., & Wengel, S. P. (1991). The Short Form of the Geriatric Depression Scale: A Comparison With the 30-Item Form. *Journal of Geriatric Psychiatry and Neurology*, *4*(3), 173–178. https://doi.org/10.1177/089198879100400310

Buysse, D. J., Reynolds, C. F., Monk, T. H., Berman, S. R., & Kupfer, D. J. (1989). The Pittsburgh sleep quality index: A new instrument for psychiatric practice and research. *Psychiatry Research*, *28*(2), 193–213. https://doi.org/10.1016/0165-1781(89)90047-4

Cohen, S. (1994). *PERCEIVED STRESS SCALE*.

Folstein, M. F., Folstein, S. E., & McHugh, P. R. (1975). 'Mini-mental state'. A practical method for grading the cognitive state of patients for the clinician. *Journal of Psychiatric Research*, *12*(3), 189–198. https://doi.org/10.1016/0022-3956(75)90026-6

Forstmeier, S., & Maercker, A. (2009). Motivational reserve: Lifetime motivational abilities contribute to cognitive and emotional health in old age. *Psychology and Aging*, *23*(4), 886. https://doi.org/10.1037/A0013602

Lezak, M. D., Howieson, D. B., Loring, D. W., Hannay, H. J., & Fischer, J. S. (2004). *Neuropsychological assessment* (4th ed.). Oxford University Press.

Peterson, N. G., Mumford, M. D., Borman, W. C., Jeanneret, P. R., & Fleishman, E. A. (1999). An occupational information system for the 21st century: The development of O*NET. *American Psychological Association*. https://doi.org/10.1037/10313-000

Wechsler, D. (2008). Wechsler Adult Intelligence Scale--Fourth Edition. *Archives of Clinical Neuropsychology*.

Wilson, R. S., Barnes, L. L., & Bennett, D. A. (2003). Assessment of lifetime participation in cognitively stimulating activities. *Journal of Clinical and Experimental Neuropsychology*, *25*(5), 634–642. https://doi.org/10.1076/jcen.25.5.634.14572

Winer, J. R., Mander, B. A., Helfrich, R. F., Maass, A., Harrison, T. M., Baker, S. L., … Walker, M. P. (2019). Sleep as a Potential Biomarker of Tau and β-Amyloid Burden in the Human Brain. *Journal of Neuroscience*, *39*(32), 6315–6324. https://doi.org/10.1523/JNEUROSCI.0503-19.2019

Youn, J. C., Kim, K. W., Lee, D. Y., Jhoo, J. H., Lee, S. B., Park, J. H., … Woo, J. I. (2009). Development of the Subjective Memory Complaints Questionnaire. *Dementia and Geriatric Cognitive Disorders*, *27*(4), 310–317. https://doi.org/10.1159/000205512
